# Effect of climate change on the health and nutritional status of children and their families in Africa: Scoping review

**DOI:** 10.1371/journal.pgph.0004897

**Published:** 2025-07-14

**Authors:** Mutshidzi Mulondo, Adam Hege, Joyce Tsoka-Gwegweni, James Ndirangu

**Affiliations:** 1 Division of Public Health, Faculty of Health Sciences, University of the Free State, Bloemfontein South Africa; 2 Department of Public Health & Exercise Sciences, College of Health Sciences, Appalachian State University, Boone, North Carolina, United States of America; 3 The Joint United Nations Programme on HIV/AIDS (UNAIDS), Pretoria, South Africa; Boston University Chobanian and Avedisian School of Medicine, UNITED STATES OF AMERICA

## Abstract

The health and nutritional status of children and their families is essential particularly during climate change. Most of the Sustainable Development Goals (SDGs) affect children in some way, namely, poverty (SDG 1), hunger (SDG 2), health (SDG 3), climate change (SDG 13). Evidence suggests that most countries are behind in achieving the SDGs, with only 17% of the SDGs currently achieved. The reason is because the SDGs are interconnected such that failure in one SDG, may affect the others negatively. For example, evidence from the global north provides many examples of the effects of climate change on other SDGs, particularly health. Within the global south, evidence of the effects of climate change on health is limited. This scoping review aims to document the effects of climate change on the health and nutritional status of children and their families in Africa. The review was conducted according to the Preferred Reporting Items for Systematic Reviews and Meta-Analyses extension for Scoping Reviews (PRISMA-ScR). Three electronic databases were searched by a librarian. One reviewer screened the articles to be included in the synthesis and a second author went through the selected articles to confirm their inclusion. Data was extracted and mapped according to four categories: i) climate change events or phenomena, ii) effect of climate change on nutritional and health status, iii) factors influencing vulnerabilities of population to climate change, iv) interventions and innovations used to mitigate impact of climate change on health.

## Introduction

The Lancet Climate Commission concluded that anthropogenic climate change threatens to undermine the past 50 years of gains in public health through associated disasters like droughts, floods, temperature changes, and changing vector patterns among others [1]. Reports indicate that children pay an unequal price for climate change with an estimated up to 88% of the disease burden related to it [2]. Therefore, climate change could magnify the already existing vulnerabilities of children and other populations at risk and could considerably obstruct future progress and possibly even reverse the advances made in child survival and well-being during recent decades [3]. The effects of climate change on child health travel through many different pathways, vary significantly across geographical locations, and are heavily influenced by broader socio-economic contexts [3].

The effects of climate change on pregnant women are beginning to be understood. Extreme weather changes during pregnancy have been associated with an increased risk of preterm birth [4,5] partly attributable to water scarcity [6] with implications for the health of the neonate and the development of the child [7,8]. Additionally, an increased risk of overall mortality from weather variability in children has been reported, particularly in infants [9,10]. Globally, malnutrition is one of the leading factors attributable to child morbidity and mortality and has been associated with lower academic performance [11]. Extreme weather events, including both flooding and drought, increase the vulnerability of subsistence farmers [12] and have long-term effects on the nutritional status of children with the most vulnerable being at the highest risk [13–14]. Reports on the association between temperature and all causes of diarrhea imply that climate change could be responsible for a substantial portion of diarrhea cases [15] partly due to unsafe drinking water [16,17].

One particular region drastically impacted by climate change is Southern Africa. Concerningly, a recent review paper highlighted major concerns regarding food security and nutritional health in Southern Africa as it relates to climate change [18]. Much of this is due to continued population growth and increased demand for food, scarcity of resources, and water shortages. With children being among the most vulnerable to climate change and the long-term implications to their health across the lifespan, as well as Southern Africa being a region of serious concern, it is important to further understand the impacts on children in the region. Extreme weather events such as Cyclone Idai in Mozambique have had an effect on child health through health services disruptions [19]. The recent increases in Cholera cases in Southern Africa surged more than four-fold in Malawi, Zimbabwe and Mozambique between 2022 and 2023 [20]. Of particular climate change concern is South Africa, which is in the southern-most point of Africa. According to the United Nations Development Plan, South Africa lies on a drought-bed which is often evidenced by low rainfall and high temperatures. Unfortunately, due to economic, logistical, and social challenges there have not been sustainable and lasting interventions. They have been smaller scale/ not far-reaching. There have also recently been floods in parts of South Africa such as Durban and Cape Town [21]. Therefore, the purpose of this scoping review was to document the effects of climate change on the nutritional and health status of children and their families in Africa. There are no similar prior reviews on this topic to our knowledge. The review attempted to achieve four objectives: 1) identify the climate change events or phenomena affecting health, 2) identify the direct and indirect effects of climate change on health and nutritional status, 3) identify the factors influencing vulnerabilities of population to the effects of climate change, and 4) report on the interventions and innovations that are recommended for reducing the impact of climate change on health. Specific topic areas reviewed include food production and agricultural systems, household food security, malnutrition, food safety, nutrition behaviour, and community-level interventions.

## Materials and methods

### Study design

To conduct the scoping review, the research team utilized Arksey and O’Malley’s [22] framework as well as further recommendations provided by Levac and colleagues [23]. This included: (1) identifying a clear objective(s) to be addressed in the review; (2) identifying relevant literature by conducting a literature search on EBSCOHost and Google Scholar electronic databases; (3) Screening the literature to be used in the synthesis and data extraction; (4) by recording the data; and (5) summarizing and reporting the findings of the synthesis and review.

### Definitions

**Nutritional Status**- The result between the nutritional intake demand which allows for the utilization of nutrients to maintain reserves and compensate for losses [24].

**Malnutrition**- According to the World health Organisation, malnutrition are deficiencies or excesses in nutrient intake, imbalance of essential nutrients or impaired nutrient utilization [25].

**Climate change**- According to the United Nations, climate change refers to long-term shifts in temperatures and weather patterns [26].

**Food security**- According to the World Bank, food security is when all people, at all times, have physical and economic access to sufficient safe and nutritious food that meets their dietary needs and food preferences for an active and healthy life [27].

### Inclusion and exclusion criteria

The PCC (Population, Concept, Context) framework by the Joanna Briggs Institute (JBI) was followed in order to illustrate the inclusion and exclusion criteria [28] (Pollock, et a., 2021). The inclusion criteria included all studies about climate change published about children and their families in Africa. All research designs were considered and included in this study.

The exclusion criteria on language was limited to English. Studies that did not include a clear methodology and results were excluded.

Inclusion criteria:

- Peer reviewed scientific and grey literature.
- Quantitative, qualitative, and mixed methods studies conducted in Africa.
- Describing impacts of climate change on food and nutritional health and related outcomes in Africa.
- Independent studies, governmental reports, systematic reviews, commentaries related to climate change impacts on health in Africa.
- Unlimited year period as there is no year limit set.

Exclusion criteria:

- Studies that were not in English.
- Studies that were conducted outside Africa.
- Duplicate studies.
- Studies that did not include health

### Search strategy

The scoping review included literature from any year as there was no year limit set. Search strategies were developed with the assistance of a librarian for Academic Search Ultimate, Africa-Wide Information, APA PsycArticles, APA PsycInfo, Applied Science & Technology Source Ultimate, CINAHL with Full Text, Communication & Mass Media Complete, Health Source: Nursing/Academic Edition, Humanities Source Ultimate, MEDLINE, Sociology Source Ultimate, Business Source Ultimate and GreenFILE. A search was conducted using the following search string: (“climat* change*” or “global warming*” or “climat* crisis” or “climat* emergenc*” or “greenhouse gas*” or “global heat*” or “temperature change*” or “severe weather*” or “catastrophic weather*” or “extreme weather*”) AND (“household vulnerabilit*” or “food secur*” or “food insecur*” or “nutrit* deficit*” or “nutrit* status*” or malnutrit* or malnourish* or “food vulnerabilit*”) AND “africa” AND ti (“climat* change*” or “global warming*” or “climat* crisis” or “climat* emergenc*” or “greenhouse gas*” or “global heat*” or “temperature change*” or “severe weather*” or “catastrophic weather*” or “extreme weather*” or “household vulnerabilit*” or “food secur*” or “food insecur*” or “nutrit* deficit*” or “nutrit* status*” or malnutrit* or malnourish* or “food vulnerabilit*”). The university librarian assisted in finalizing the key term search strategy and in obtaining documents which were not easily accessible to the researchers. The databases were last searched in August 2024 and all available literature was used until that date due to limited literature on the topic.

### Study selection

Citations were manually screened for the data extraction process. One reviewer (JN) screened the title, abstract and where necessary the full-text to determine eligible studies. Once the reviewer had completed the article selection & data extraction, JTG went through the selected articles to confirm their inclusion. All authors agreed on the inclusion and exclusion criteria, there was therefore consensus. Any conflicts were resolved with the PI (MM).

### Data extraction and analysis

The data extraction table includes author, year of publication, title, purpose, methods/design, population and sample size, country, intervention type, key findings and recommendations. The data extraction was conducted by one researcher (JN) who identified duplicates and studies which did not meet the inclusion criteria and removed them from the final synthesis. The researcher then extracted data from the included studies to create the final data extraction table (see S1 Table).

Data analysis was descriptive and consisted of summaries based on the following variables: study, study type, study method, study design, sub-region, country, setting and study population/systems.

## Results

**Fig 1:**
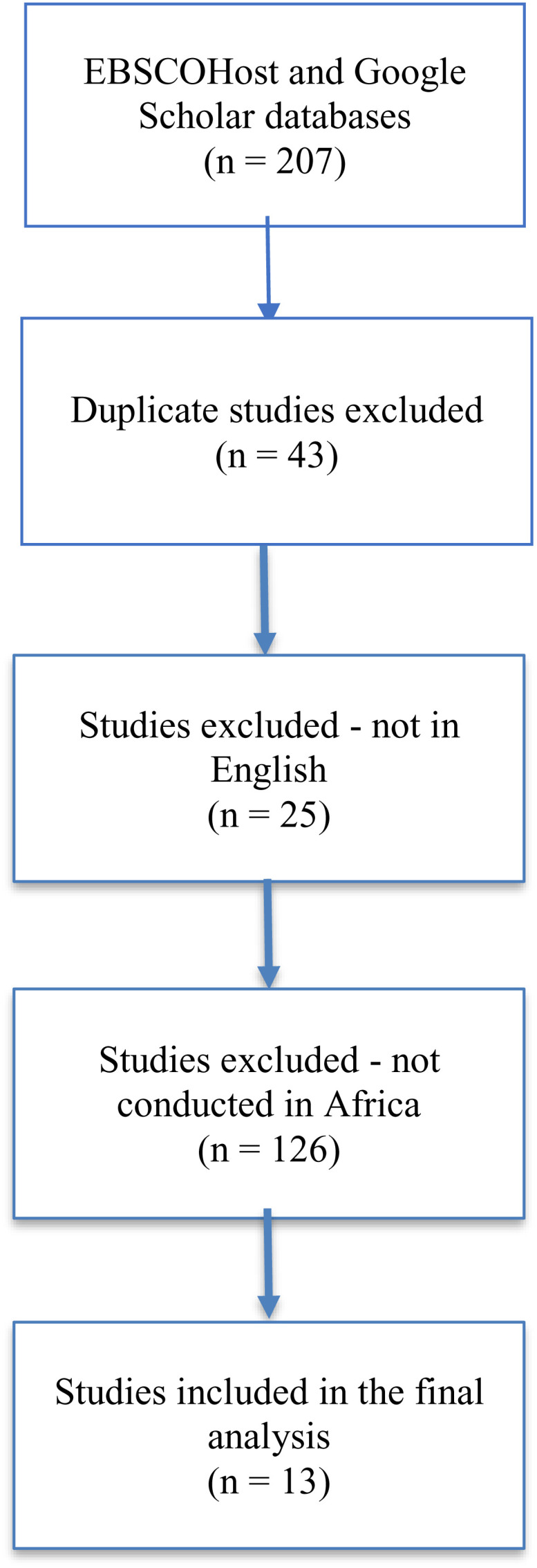
PRISMA Diagram.

In the database search, 207 titles and abstracts were identified. After screening, 17 records were included in this review. From the 17 studies, four were excluded as they did not focus on children nor health (see Fig 1).

### Study characteristics

All 13 studies included took place in Africa, dominated by Southern Africa (8/13), then followed by combined African regions, West Africa, East Africa and global including Africa. The majority of the studies were conducted in South Africa (7/13), then Kenya (1) and Nigeria (1) and the rest were mixed African countries. The type of articles included in the review were four original, five reviews and four editorial or commentary articles. Six of the articles used a qualitative approach, two quantitative and five mixed methods. The most utilized study design was descriptive (6), scoping review (3), then followed by exploratory, cohort, case study and modelling.

Most of the studies were conducted in all the areas of the countries and focused on all groups of the population. Only a few studies researched specifically children (Table 1).

**Table 1:** Study characteristics.

Study	Study type	Study method	Study Design	Sub-region	Country	Setting	Study Population/ systems
Van der Merwe et al, 2022	Original	Quantitative	Cohort	West Africa	Nigeria	Whole country	Children
Sheriff & Mash, 2022	Commentary/ Narrative review	Qualitative	Case study	East Africa	Kenya	Chakama	All ages
Erzse et al, 2023	Commentary	Qualitative	Descriptive	Southern Africa	South Africa	Whole country	All ages
National Institute of Communicable Diseases, 2022	Commentary/ Editorial	Qualitative	Descriptive	Southern Africa	South Africa	KwaZulu-Natal province	All ages
Mthethwa & Wale, 2023	Original	Quantitative	Modelling	Southern Africa	South Africa	All nine provinces	All ages
Nakstad, 2022	Review	Qualitative	Descriptive	All regions	Whole continent	All countries	Neonates
Enwereji, 2021	Original	Qualitative	Exploratory	Southern Africa	South Africa	Five municipalities	Municipal workers
Ngumbela et al, 2020	Original	Mixed methods	Descriptive	Southern Africa	South Africa	Eastern Cape province	All population groups
Wlokas, 2008	Editorial	Qualitative	Descriptive	Southern Africa	Namibia, Botswana Zimbabwe Mozambique Lesotho Swaziland South Africa	All areas in these countries	All population groups
Cherish et al, 2018	Review	Mixed methods	Scoping review	Southern Africa	South Africa	All areas	All ages
Hellden et al, 2021	Review	Mixed methods	Scoping review	Global	All countries	All areas	Children
Wright et al, 2021	Narrative review	Mixed methods	Descriptive	Southern Africa	South Africa	All areas	All ages
Lokotola et al, 2023	Review	Mixed Methods	Scoping review	All regions of Africa	All countries in Africa	All areas	PHC systems

### Effect of climate change and health, and nutritional status

The types of climate change indicated were high temperature (resulting in heat stress), drought and heavy rainfall or flooding, air pollution and wildfires. These climate change events may affect people’s health either directly or indirectly. A number of factors or vulnerabilities of people increase the risk of people’s health and nutritional status being affected by these climatic conditions or events (Table 2).

**Table 2:** Effects of climate change on health and nutritional status of children and their families.

Climate indicator	Outcome	Factors	Population
High Temperature (Heat Stress)	Malnutrition - high levels of stunting and underweight Morbidity and mortality (cardiovascular and renal diseases) Vector-borne disease Fetal heart problems Reduced foetal movements Meconium aspiration syndrome Jaundice Heat exhaustion Neurological dysfunction Dehydration Hospital admissions Peripheral gangrene Convulsions Preterm birth Low birth weight Stillbirth, miscarriages, birth defects, pneumonia, atopic eczema Poor nutrition and breastfeeding Affected growth development	Poor food quality resulting in food insecurity Decline in milk production Pregnancy complications Ecosystem disruption Cultural, social determinants, poverty (loss of livelihoods) Lack of social security (grants) High costs of health	All Children Neonates Pregnant women

#### High temperatures.

From the reviewed studies, the effects of high temperatures and heat stress are associated with a number of health problems and nutritional status for pregnant women, neonates and children, (especially under five-year-olds). Among pregnant women, the effects include increased gestational hypertension and diabetes, and pre-eclampsia during labour, poor nutrition and breastfeeding, pneumonia, fetal heart problems and reduced fetal movements. Further effects include adverse pregnancy outcomes (stillbirths, preterm delivery, miscarriages, low birth weight babies), vector-borne and cardiovascular diseases. Among neonates and children the effects recorded include; stunting and undernutrition, dehydration, hospital admissions, increased risk of morbidity and mortality from respiratory, cardiovascular and vector-borne diseases. Extreme solar UV radiation is associated with increased risk of skin cancers. High humidity could also result in heat related illnesses such as dehydration, heat exhaustion and heat-stroke (Table 2).

#### Drought.

The effects of drought include mental health problems, trauma and substance abuse in adults. Due to shortage of water for household consumption and food production, all population age groups are at risk of food insecurity (malnutrition). This also increases the vulnerability of women and girls to sexual violence, exploitation and HIV. Other factors that increase people’s risk or vulnerability to malnutrition are unemployment and death of livestock in the farming sector as a result of drought. Similarly, drought linked to poor general health, contaminated water and poor hygienic conditions are a risk for food-borne and vector-borne infections such as cholera, malaria and schistosomiasis. Drought has also been found to be associated with stress, mental health, intimate partner violence, and food insecurity. Stresses in pregnancy and perceived stress in general, are associated with preterm birth [29] (Tanpradit and Kaewkiattikun, 2020). Drought has also been associated with a danger of being attacked by wild animals as they visit people’s homes in search of water. As a result of climate change related drought, people may decide to migrate to areas with water or near rivers. In most cases migration may create further health and livelihood challenges for people in both transit and host communities (Table 2).

#### Floods.

The effects of floods include, drowning, unintentional injuries, stunting and underweight and high mortality rates in children and adults, long term effects on child health due to lack of proper nutrition, and communicable diseases such as malaria and respiratory diseases. Floods and too much rain could ruin crops or make harvesting impossible, thereby reducing food access and availability. Anemia in the third trimester has been found to be associated with low birth weight. The excess rainfall/floods could result in anemia through reduced intake of iron-rich foods [30] (Diamond-Smith, et al., 2023). Other effects identified include mental health in adults, skin infections, food-borne and water-borne infections, respiratory infections because of poor nutrition and hygienic conditions, damage to water supply and healthcare infrastructure and overcrowded living conditions of displaced people. (Table 2).

#### Air pollution.

Air pollution is associated with a risk of respiratory diseases, chronic obstructive pulmonary disease, heart disease, vector-borne diseases due to poor air quality and pollution. This also affects lung function. Air pollution is also linked to mental health problems in adults, emotional and psychological distress, hospital admissions and even death. In pregnancy, air pollution is associated with adverse pregnancy outcomes like LBW and small for gestational age (Table 2).

#### Wildfires.

the negative impact of wildfires include malnutrition due to a threat on food security. The direct effects include smoke inhalation, destruction of agricultural and ecological systems and damage to crops, livestock or harvested products. Smoke inhalation may lead to respiratory diseases, while land and infrastructure damages may lead to displacement and social interruption (Table 2).

### Socio-economic, demographic factors influencing vulnerabilities of populations to climate change

Table 2 further indicates certain factors that influence people’s vulnerabilities, which may act as enablers of the effects of climate change. For example, food insecurity increases the risk of malnutrition as reported that households in uMzinyathi District of KwaZulu-Natal were found to be severely food insecure. Also the impacts of food security were reported to be more severe in rural areas than in urban locations. Similarly, it was observed that limited resources were available for neonates. Another example are the effects on agricultural processes. Evidence shows that farmers are lacking critical resources and information that could be useful in their processes. These barriers include resources and the financial limitations. Limited water quantity and quality, livestock production decline, negatively impacting beef and dairy industry necessary for ensuring good nutritional and health status.

In terms of factors influencing vulnerabilities among neonates, children, pregnant women and women in general. The effects of climate change and other stressors are reported to be much more severe in these groups. It is reported that households with a female-head are at a higher risk of food insecurity threatening their nutritional and health status. In addition, gaps between women and men, specifically households with a woman as head, are more severe in rural areas when compared to urban settings. Other reported factors exacerbating vulnerabilities of people include poverty, limited financial resources and elderly people, lack of access to health infrastructure and services especially for neonates and pregnant women, make people more vulnerable to the effects of climate change as the sick cannot access health services.

### Interventions to address the effects of climate change on health

Recommended interventions to address the effects of climate change on nutritional and health status are grouped into mitigation and adaptation. Examples include health education and awareness programmes, climate-smart agriculture such as the use of organic matters to prevent food insecurity and climate-friendly policies like reducing the effect of carbon emission and energy-conserving policies.

Farmers adaptation strategies include: different crops and varieties; planting of trees, soil conservation and irrigation. Implementing nature based and innovative water storage and management systems, which can play a crucial role in mitigating water scarcity during prolonged dry spells, ensures a more reliable and sustainable water supply for farming.

## Discussion

The bulk of the evidence on the impact of climate change on health and nutritional status of children and their families is found in resource-rich settings. Very little evidence exists in resource-poor settings especially within the African continent. The few studies included in the current review were mainly reviews (Cherish et al, 2018; Hellden et al, 2021; Wright et al, 2021; Lokotola et al, 2023; Nakstad, 2022), used qualitative and mixed approaches (Sheriff & Mash, 2022; Erzse et al, 2023; NICD, 2022; Nakstad, 2022), were conducted within the Southern African region (Cherish et al, 2018; Erzse et al, 2023; NICD, 2022; Mthethwa & Wale, 2023; Ngumbela et al, 2020; Wlokas, 2008; Wright et al, 2021) and focused on all ages of the population (Cherish et al, 2018; Erzse et al, 2023; Mthethwa & Wale, 2023; NICD, 2022; Sheriff & Mash, 2022). The most common climate change events or phenomena were high temperatures, drought and floods. The findings agree with those reported elsewhere about the same climate change events were (Hellden et al, 2021; UNICEF, 2024) [31].

The effects of high temperatures on nutritional status and health of pregnant women, neonates and children have been reported in other countries as the major contributor to morbidity and mortality among this group (Zhao et al, 2019). The findings from this review also agree with those reports. This could also explain the reason why Africa has high neonatal, child and maternal morbidity and mortality (UNICEF, 2024) [31]. Similarly, the impact of drought and floods are stunting and underweight, infections and mental health as a result of food insecurity, damaged infrastructure, food-borne and waterborne infections, poor hygienic conditions. This is not a surprise as drought and floods have become common phenomena in recent years affecting many parts of Africa including Southern Africa (UNHCR, 2023; WFP, CPD, 2024). Currently, South Africa has been severely affected by floods, drought and extremely high temperatures. In addition to the devastation to livelihoods, there is a threat of emerging and re-emerging infectious diseases (NICD, 2022). The effect of extreme weather events on child health is indirect, through the various food channels. A study in Uganda found that weather extremes that were studied had a negative and statistically significant effect on caloric supply (i.e., on protein, zinc, and vitamin A supply). The dry spells in the previous season led to a reduced protein and zinc supply by 37% and 28%, respectively (Armondo, et al. 2023). Many risk factors identified to be influencing the vulnerabilities of people to the effects of climate change in Africa are related to socio-economic and demographic factors such as age, gender, poverty, household income and financial security, household head, region, food insecurity, physical infrastructure, access to clean water and access to health services (Ncube, 2016; Ngumbela, 2020; NICD, 2022; Tibesigwa, 2016; Byan, 2006; Nyoni, 2022; Enwereji, 2021; Muyambo 2023; Zwane, 2019; Mafongoya, 2016; Nakstad, 2022).

Since, literature is full of many studies reporting on the effect of climate change on food security within the agricultural sector (Enwereji et al, 2021; Zwane 2019; Ngumbela et al, 2020; Nyoni et al, 2022; Muyambo, 2023; Mthethwa & Wale 2023), there is a need for further empirical research within African settings providing a better understanding between climate change and nutritional and health status. South Africa is off-track when it comes to the Sustainable Development Goals (SDGs), namely SDGs 2 and 3. About 23% of children in South Africa are at risk of life-threatening malnutrition, which remains one of the highest in the world, and related health complications (UNICEF, 2024) [31]. The proportion of households in South Africa that experienced moderate to severe food insecurity was estimated at 15,8% in 2019, 16,2% in 2022, and 19,7% in 2023. The percentage of households engaged in agricultural activities and facing moderate to severe food insecurity in 2023 was estimated to be 25,5%, while the percentage of households not involved in agriculture was surprisingly lower at 18,5%. Approximately, one in four households that resided in traditional areas were affected by moderate to severe food insecurity in 2023 which was also the highest across all settlement types (Stats SA, 2025) [32].

Sub-Saharan Africa has become common place for extreme weather and climate change conditions affecting health, but there is limited documented evidence. Understanding the effects of climate change on health will require collaboration of multi-stakeholders, multi-disciplines and multi-sectors across the various African settings, because one climatic disaster in one country or sector may result in severe impact on the rest of all other sector in a country and neighbouring countries like we have witnessed with the spread of COVID-19 pandemic, Monkey-pox, malaria, measles and other infectious diseases (IPCC, 2023; SADC report; WHO AFRO report; AU report).

Though there are a number of reports on innovative interventions developed to mitigate and adapt against the effects of climate change on food security in Africa (Mafongoya, 2016; Bryan, 2006; WFP, 2023; Zhao et al, 2022; Kim et al, 2023; Redvers et al, 2023), these studies have not focused on nutritional and health status of populations especially African children.

### Recommendations

Decision and policy-makers need to prioritize the needs of vulnerable populations including children, women and those in rural areas. This means designing and implementing policies and programmes that target those most at risk of being overlooked and ensuring they have equitable access to resources, healthcare, support systems and opportunities. Additionally, sustainable financing is critical for addressing the environmental and climate determinants of health, and for building climate resilient health systems. Currently, funding for climate action and for tackling the climate–health nexus is inadequate when compared to state funding of carbon producing industries. Further, it is critical to develop climate resilient water supply and sanitation systems designed with climate resilience in mind, while also retrofitting existing systems. Lastly, promoting community engagement that includes the roles women and youths can play in climate change adaptation, disaster preparedness, response, and recovery is important for local ownership.

Lastly, we recommend research that will generate empirical evidence of the effect of climate on nutritional and health status of children and adults within the Africa settings in all regions. We further recommend research that can explore the interventions and innovative ways of averting the negative effects of climate change on nutritional and health status of African children and adults.

## Conclusions

Even though Africa is known to carry the highest burden of disease and is vulnerable to extreme climate change events, this review shows that there is limited data on the effects of climate change on nutritional and health status of the populations in African settings. There is a lack of empirical data. The limited evidence found consists mainly of reviews from Southern Africa. The most commonly reported adverse climatic change phenomena or events facing the African continent currently are high temperatures, drought and floods. A few studies also reported on wildfires and air pollution. The findings from this review confirm the main health conditions associated with the climate change events are malnutrition, infectious diseases, respiratory diseases in children and adults, adverse pregnancy and birth outcomes, high child and maternal morbidity and mortality, and mental health problems. The identified vulnerabilities increasing the risk of people to the effects of climate change events on poor nutritional and health status include personal and socio-demographic, economic and environmental factors. the effects of these climatic change phenomena on nutritional and health status of children and adults and some of the related factors that make populations more vulnerable to such climatic factors.

There available and innovative approaches that have proven successful in mitigating and adapting to the effects of climate change are mainly focusing on the agricultural sector. Some of these can indirectly avert the negative impact on health status.

## Supporting information

S1 Table Appendix 1. (DOCX)
