# Supplementary material for: Effect of climate change on the health and nutritional status of children and their families in Africa: Scoping review
Source: PLOS Glob Public Health. 2025 Jul 14;5(7):e0004897. doi: 10.1371/journal.pgph.0004897 (PMC12258551; doi:10.1371/journal.pgph.0004897)
Supplement: S1 Table — (DOCX) [file pgph.0004897.s001.docx]

**Appendix 1**

Table 1: Data Extraction Table

| **Author** | **year** | **title** | **purpose** | **Methods / design** | **Population & sample size** | **country** | **Intervention type (if applicable)** | **Key findings** | **recommendations** |
| --- | --- | --- | --- | --- | --- | --- | --- | --- | --- |
| Shackleton, S | 2014 | A gendered perspective of vulnerability to multiple stressors, including climate change, in the rural Eastern Cape, South Africa | the gendered implications of multiple stressors on livelihoods | A household survey, interviews and focus group discussions | four-year research project on household livelihoods | South Africa |  | Women and female-headed households are generally poorer and more at risk than men and male-headed households. In some situations women may be more innovative in their individual and collective responses to stressors and may have more social capital to draw on | There is a need to better understand the underlying causes of vulnerability and the heterogeneity that exists at the local level. |
| Mafongoya | 2016 | Adaptation to climate change and the impacts on household food security among rural farmers in uMzinyathi District of Kwazulu-Natal, South Africa | Examines the methods of adaptation to climate change used by smallholder farmers and their impacts on household food security | Household surveys | smallholder farmers | South Africa |  | Households undertake crop and soil management practices in order to respond to the changing climate. Overall, 97% were severely food insecure and the remaining 3% were moderately food insecure | Information will play a critical role in mitigating the impacts of climate change on household food security but farmers should also be assisted with appropriate input packages, such as seeds and fertilizers that can help them adapt effectively |
| Bryan, Elizabeth | 2006 | Adaptation to climate change in Ethiopia and South Africa: options and constraints | understanding of farmers’ perceptions of climate change, ongoing adaptation measures, and the decision-making process | Survey of 1800 farm households | farmers | Ethiopia and South Africa |  | Most common adaptation strategies include: use of different crops or crop varieties, planting trees, soil conservation, changing planting dates, and irrigation.  SA - The main barriers to adaptation cited by farmers were lack of access to credit. | Policy-makers must create an enabling environment to support adaptation by increasing access to information, credit and markets, and make a particular effort to reach small-scale subsistence farmers (with limited resources). |
| Tibesigwa, Byela | 2016 | Assessing Gender Inequality in Food Security among Small-holder Farm Households in urban and rural South Africa | Assessing gender inequality in household food (in) security | Survey | Small-holder farm households in urban and rural areas | South Africa |  | Male-headed households are more food secure compared to female-headed households, with the latter depending more on agriculture to increase household food levels.  The security gap is wider in rural than in urban areas with higher risk to report chronic food insecurity in rural than urban areas. | Align food security initiatives with policies on urban and rural agriculture and development. |
| van der Merwe, Eduard | 2022 | Climate change and child malnutrition: A Nigerian perspective | the effect of climate change (changes in the monthly maximum average near-surface temperature and total monthly precipitation) on children's health outcomes | Secondary data (gridded climate data & Living Standards Measurement Study) | children | Nigeria |  | We find that the rise in temperature is associated with higher levels of stunting in children – even more so in rural areas. | The need for climate-friendly policies to mitigate the long-term effect of climate change on malnourishment |
| Sheriff, Muhsin | 2022 | Climate change and primary health care in Chakama, Kilifi County, Kenya | Evaluate how climate change affects primary health care services. |  | PHC level | Kenya | Chakama is an area of 46 small villages in Kilifi County | The health effects are seen in the rising number of people suffering from malnutrition and gastroenteritis as well as in terms of mental health problems.  Facilities and healthcare workers also struggle to be resilient in the face of the same environmental challenges | Support required includes access to health and social services, community engagement and multisectoral action. |
| Ncube M | 2016 | Climate change, household vulnerability and smart agriculture: The case of two South African provinces | To explore the impact of climate change at the local level, especially in rural areas. | Household level |  | South Africa | This study looked at the micro-level impact of climate change, evaluated household vulnerability and assessed alternative adaptation strategies in rural areas | Climate change will hit crop yields hard and that households (HH) with less capital are most vulnerable. These HH consist of the elderly and households headed by females.  HH that receive remittances or extension services or participate in formal savings schemes in villages are less vulnerable. | HH need to move towards climate-smart agriculture, which combines adaptation, mitigation and productivity growth |
| Muyambo F | 2023 | Climate-Change-Induced Weather Events and Implications for Urban Water Resource Management in the Free State Province of South Africa | Climate-change-induced weather events and the implications for urban water resource management in South Africa | Literature review  (122 documents which include books, peer-reviewed articles, conference papers, newspaper articles, institutional and government reports, and one news broadcast video) | Particularly focusing on QwaQwa | South Africa |  | Increasing water challenges as demand for water increases and both quantity and quality decrease to critical levels | Conducting hazard, exposure, and resilience analyses is necessary in order to inform the development of relevant disaster risk reduction strategies |
| Agnes Erzse et al | 2023 | Commentary on South Africa’s syndemic of undernutrition, obesity, and climate change | Commentary focuses on how the Global Syndemic manifests in South Africa, and on one of its major common drivers, the food system | Commentary |  | South Africa |  | With increasing malnutrition and worsening climate, costing billions of rands annually, this is an opportune time to review the drivers of these major challenges and search for comprehensive and efficient approaches in tackling complexities of the syndemic. The causes of these issues are not singular in nature as they arise from several issues including pricing, marketing, and the availability of nutritious food | Policy options available to simultaneously tackle the syndemic include the breaking down of silos of action, improving the collection and utilisation of data, scaling up nutrition financing, focusing on healthy diets in the systems, and improving the governance structure for actors in the food system. |
| National Institute for Disease Control | 2022 | Health risks associated with flood disasters | KZN | Literature review |  | South Africa |  | NICD identified five health risks arising from the flooding:  1.Acute events such as drowning  2. NCDs due to non-adherence; lack of access of health services; damage to health infrastructure; mental health; increases risk of infections |  |
| Mthethwa, Sandile & Wale E | 2023 | Household vulnerability to climate change in South Africa: A multilevel regression model | To measure the vulnerability of households to food insecurity by measuring the risk or threat posed by climate change | Multilevel or hierarchal regression | Sample size of 18,444 households nested within nine provinces |  |  | Climate change poses serious threats that expose households to future food consumption inadequacies.  Poverty or structural-induced vulnerability emerged as the main source of vulnerability for South African households with rural areas being more vulnerable. |  |
| Nakstad, Britt | 2022 | How Climate Change May Threaten Progress in Neonatal Health in the African Region |  | Literature review | low-resource settings | Africa |  | Neonates are at high risk of heat stress and dehydration due to their unique metabolism, physiology, growth, and developmental characteristics. Climate change may increase risks to neonatal health from weather disasters, decreasing food security, and facilitating infectious disease transmission. | Effective interventions to reduce risks from the heat include health education on heat risks for mothers, caregivers, and clinicians; nature-based solutions to reduce urban heat islands; space cooling in health facilities; and equitable improvements in housing quality and food systems. |
| Enwereji, P. C | 2021 | Impact of Climate Change on Food Security and Water Supply in South Africa | This study explores the impact of climate change in the local municipalities of South Africa and the possible sustainability strategies to ensure that development is resilient to climate change. | Qualitative research approach and an exploratory research design has been used | Sixty-five (65) municipal employees, including health and agricultural employees | South Africa |  | Climate change has numerous impacts on the livelihoods of South Africans, which has impacted negatively on their developmental pace.  The demand for water has increased, while its supply has drastically reduced due to evaporation. The arable land has frequently decreased due to dearth of rainfall | The study recommends that local authorities should improve the climate risk considerations into meaningful policies to guarantee positive development in water supply and food security |
| Zwane EM | 2019 | Impact of climate change on primary agriculture, water sources and food security in Western Cape, South Africa | To assess the impact of climate change on primary agriculture and food security. | A literature review | 11 government reports and 21 journal articles including experience outside Western Cape | South Africa |  | Many dams had low water levels (40%) during 2016/2017, which reduced crop yields including grapes. Droughts, which affected both smallholder and commercial farmers, are now a common phenomenon. Livestock production has declined over time, with small stock, the beef and dairy industry being the most affected | The major recommendations included scaling up on the use of organic matter to avoid burning and creating gas emissions to the atmosphere, the effective use of livestock manure and the use of appropriate and adaptable seed varieties |
| Ngumbela XG et al | 2020 | Vulnerability and food insecurity in the Eastern Cape province of South Africa | This article examines the extent to which the EC can be defined as vulnerable to food insecurity | Review of current literature |  | South Africa |  | The elderly and children are affected by life cycle vulnerability factors, with children prone to malnutrition and the elderly unable to work to produce food. Most of the people in the EC who are poor and are African, and a high percentage of women-headed households is poor. | Interventions need to take local contexts into account and focus on particular communities and their unique needs. |
| Wright CY  *(refer to the full text pdf attached)* | 2021 | Major climate change-induced risks to human health in South Africa | This article aims to describe the major climatic changes facing South Africa and how they can impact human health. | Review of literature |  | South Africa |  | An increase in ambient temperature, causing, for example, a significant rise in morbidity and mortality; heavy rainfall leading to changes in the prevalence and occurrence of vector-borne diseases; drought-associated malnutrition; and exposure to dust storms and air pollution leading to the potential exacerbation of respiratory diseases; the incidence of skin cancer may  also rise. | Whether changes in the climate will result in  severe health issues for South Africa will depend largely on the provision  of better living and socioeconomic conditions as outlined in the National  Development Plan 2030, in conjunction with improved policies and  mitigation interventions.  Adequate education regarding personal pro-  tective behaviour, vector control and ready access to medical care and  treatment will also be important. |
| Nyoni, Njongenhle M. B. | 2022 | Perceived impacts of climate change on rural poultry production: a case study in Limpopo Province | We aim to establish farmers' perspectives on likely impacts of climate change on their rural poultry production in northern South Africa. | A baseline questionnaire-based study | 106 households | South Africa |  | Most households lacked reliable and adequate sources of income and had, for example, days when they had to skip meals as a coping strategy. They had poor access to scientific information on agricultural production. Farmers reported a reduction in poultry productivity in recent years, coinciding with increased ambient temperatures. | The farmers in our study perceived their poultry as hardy and well adapted to survive any future climatic changes and may be uninclined to take adaptive action at this stage. |
